# Improved Chronic Low Back Pain, Radiographic Alignment, and Patient Reported Outcomes Following Postural Rehabilitation Protocols: A Case Series of Two Patients with 18- and 26-Months Follow-Up

**DOI:** 10.3390/healthcare14111586

**Published:** 2026-06-04

**Authors:** Miles O. Fortner, Jason W. Haas, Thomas J. Woodham, Paul A. Oakley, Deed E. Harrison

**Affiliations:** 1 Western Plains Chiropractic, Gillette, WY 82718, USA; mofortner@gmail.com (M.O.F.); tjwoodham12@gmail.com (T.J.W.); 2 Chiropractic BioPhysics NonProfit, Inc., Eagle, ID 83616, USA; drdeed@idealspine.com; 3 Independent Researcher, Newmarket, ON L3Y 8Y8, Canada; docoakley.icc@gmail.com

**Keywords:** chronic low back pain, sagittal balance, lumbar lordosis, spine alignment, non-specific CLBP, case series

## Abstract

**Background/Objectives:** We describe a case series of two patients with non-specific chronic low back pain (CLBP) and measurable decreased quality of life, who showed improvements after a specific multi-modal conservative spine and postural therapy regimen. CLBP is the leading cause of years lived with disability and disability-adjusted life years. This case series adds observational data to the medical literature on conservative treatment of CLBP and potentially improves diagnostic and treatment understanding of how conservative therapies can benefit patients suffering with CLBP. **Methods:** Two patients (Patient A: 58-year-old female; Patient B: 43-year-old male) presented with severe CLBP who did not find relief with prior traditional chiropractic manipulation. The patients sought treatment at a spine rehabilitation facility closest to their remote locations in Wyoming, USA. The conservative rehabilitation treatment program consisted of multi-modal therapies to strengthen postural muscles, postural spinal manipulation, and specific Mirror Image^®^ traction. After 36 treatments over 12 weeks in office and home rehabilitation exercises, baseline tests and outcome measures were repeated. **Results:** Patient-reported objective outcomes, disability indices, and radiographic analysis demonstrated changes at the conclusion of treatment that were maintained at long-term follow-up re-examination. Lumbar lordosis initially changed from −21.8° L1–L5 lordosis to post-treatment −33.6° for patient A and from −22.6° to −42.4° for patient B. Long-term follow-up demonstrated continued resolution of initial symptoms and maintained spine alignment. **Conclusions:** In these two patients, the described multimodal conservative program was associated with sustained improvements in symptoms, function, and radiographic parameters. This case series adds to prior biomedical literature regarding potential conservative interventions for treating CLBP and abnormal posture. Larger randomized controlled studies are required to evaluate generalizability and relative effectiveness.

## 1. Introduction

Here, we present a case series of two patients reporting the observed improvements in non-specific chronic low back pain (CLBP), progressive disability, and suffering for several years. The patients received prior traditional chiropractic manipulation. The treatment did not demonstrate measurable improvements in pain or objective findings. Thereafter, they received a specific therapeutic approach with the Chiropractic Biophysics^®^ (CBP^®^) technique. CLBP is a major contributor to the global burden of disease (GBD) and is currently rated the greatest cause of disability world-wide [1]. Annual estimates report treatment costs over USD 40 billion/year; equating to more than USD 2000 per patient per year globally [2]. The burden of suffering and the general fiscal burden meaningfully impacts years lived with disability (YLDs) as well as disability-adjusted life years (DALYs) [3]. CLBP is an important research topic for healthcare researchers to find strategies that potentially offer safe, easy-to-implement, and economical treatment protocols.

Abnormal measured spinal parameters and altered postural alignment contribute to CLBP. Current investigations have demonstrated that specific conservative therapies targeting the altered alignment with Mirror Image^®^ (MI^®^) exercise, specific spinal manipulation, and MI^®^ postural exercise can reduce abnormal spine biomechanics [4].

This case series showing observed changes using a biomechanical approach for the assessment and treatment of CLBP adds descriptive data to treatment options. Thus, the aim of this two-person case series is to share the documented treatment protocol of two patients with chronic spine pain using methods previously examined in the literature [4,5,6]. The Chiropractic Biophysics^®^ (CBP^®^) protocol used on these patients has previously been described in the literature and will be outlined in detail in Section 2. This case series outlines details of the patients’ history and subjective pain complaints, baseline objective assessments incorporating radiographic imaging and postural evaluations, as well as the outcome measure questionnaires administered during the initial assessment, subsequent re-evaluations, and extended long-term follow-up. This treatment protocol can enable physicians to have the ability to treat patients in-office and provide treatment alongside home-based therapies and exercises that can continue to reinforce patient-specific postural and structural changes. The changes are patient-specific, and conclusions cannot be drawn from a two-person case series.

The multi-modal treatment protocol differs from traditional physical therapy in that the muscles are not simply strengthened uniformly in all directions; instead, biomechanical radiograph and postural mensuration results allow the physician to generate prescriptive recommendations which are patient-specific. In this small case series, the treatment recommendations were economical and carried a lower risk profile than those frequently associated with invasive interventions, surgery, as well as the side effects and potential addiction concerns with conventional pharmacological interventions for pain [7,8].

This case series is noteworthy for the sustained long-term results achieved with minimal additional in-office treatment following the initial in-office MI^®^ postural exercises, postural adjustments, and traction therapeutics. Both patients resided in a rural area that made repeated travel to the facility for treatment logistically difficult. As a result, care involved a brief in-office phase followed by a home treatment regimen with long-term follow-up. This approach may be relevant for patients in rural areas who cannot travel for in-office care for a long term due to physical distance, weather, etc. The report addresses outcomes from an economical in-office and home-based model suitable for individuals with geographic or resource limitations. As the case series of two patients is extremely small, no conclusions regarding the treatment efficacy are possible. The research hypothesis of this case series was that the specific multimodal CBP^®^ protocol, centered on restoration of lumbar lordosis through Mirror Image^®^ postural rehabilitation, would improve radiographic spinal alignment, and reduce chronic low back pain.

## 2. Methods

### 2.1. Ethical Statement

This benign, non-invasive, case series is exempt from institutional review board approval because of its retrospective design, in line with the Common Rule exemption under 45 CFR 46.104. Patients provided informed consent before any evaluation, examination, diagnosis, or treatment. They signed consent for publication prior to the article’s release for investigation by the authors. All identifiable patient information was removed, rendering the Declaration of Helsinki inapplicable to this simple case series. The case series was carried out with the utmost ethical standards, ensuring that patient data usage involved no risks while contributing to a deeper insight into non-invasive, non-surgical, non-pharmacological spinal rehabilitation and pain management strategies.

### 2.2. Patient A—History, Examination Results, Subjective and Objective Findings

A 58-year-old female (height 157.5 cm, weight 57.6 kg) suffering with frequent, moderate CLBP presented to a rehabilitation facility in Wyoming. She reported a history of pain in the low back and sciatica for over 10 years following a horse injury wherein the horse fell on her. She was seeking treatment for her worsening dysfunction and disability due to the pain. She reported bilateral iliotibial band tightness, frequently having cold feet and hands, and a history of periodic migraines that were associated with tinnitus. Frequent constipation was reported. She also reported occasional heart palpitations with stress, some neck stiffness, and occasional difficulty swallowing. The patient was active and lived in rural Wyoming, making travel for treatment difficult.

The patient underwent a thorough history, physical examination, orthopedic and neurological tests, and subjective and objective measures. Dermatomal testing revealed hypoesthesia at the S1 dermatome on the right. Left and right torso lateral bending range of motion (ROM) testing was positive for an increase in low-back and buttock pain on the right. Flexion and extension of the lumbar spine caused a reported increase in back pain from the right scapular region to the right posterior lumbo-pelvic spine. Cervical compression testing increased mild cervical pain with maximal compression positive for pain to the right side. Visual postural assessment showed forward sagittal balance. Patient-reported outcomes (PROs) were provided by the patient for the spine, general health, pain, and disability due to the condition. The revised Oswestry low back pain disability index (RODI) questionnaire was completed to determine the impact of her CLBP on daily life and to assess her self-rated disability likelihood from common activities like walking, sitting, or lifting [7]. Her baseline RODI score was 34%, revealing moderate-to-severe disability from CLBP (Table 1). The patient completed a 36-question health status questionnaire (SF-36) [9]. This form measures health-related quality of life (HRQoL) to compare overall health to population norms and averages. Baseline SF-36 scores measured below normal values indicated a demonstrable impact of the chronic pain on her quality of life (Table 2). A headache disability index (HDI) questionnaire was administered due to the history of migraine headaches (Table 1) and measured 56/100. Quadruple Visual analog scale (QVAS) was executed at baseline for the cervical, thoracic, and lumbar spine contribution to evaluate pain. Lumbar QVAS measured 40/100 composite [10,11].

Headache disability index (HDI) was performed and measured 26/52 for the emotional component, 30/48 for the functional component, with a total disability score of 56/100 rated for the migraines that were 1–2 times per month, and worsened CLBP indicating significant dysfunction (Table 1). Quadruple Visual analog scale (QVAS) was performed at baseline for the cervical, thoracic, and lumbar spine contribution to disability due to pain, rated as current pain, average pain, pain at best, pain at worst, and a total composite score of the responses, with the total for the thoracic spine at 20/100, indicating disability due to mild-moderate pain at baseline. Lumbar QVAS measured 3/10 at initial and overall, 40/100 composite.

### 2.3. Patient A Radiographic Findings

Digital radiography of the patient’s upright full spine in the neutral position was acquired. Anterior–posterior (A–P) and lateral radiographs were digitized with radiograph spine parameter biomechanical software designed by PostureRay^®^ (PostureCo, Inc., Trinity, FL, USA, version 23.7) [12]. The program measures parameters using the Risser–Ferguson mensuration for the AP views and uses the Harrison posterior tangent method (HPTM) to assess intersegmental and global sagittal balance for the lateral views [13]. The landmark digitizing software evaluates the patient’s spine parameters of intersegmental and global (cervical, thoracic, lumbar) measurements in the coronal and sagittal planes and compares to reliable models of ideal spine parameters [14,15]. A recent investigation found PostureRay^®^ to have remarkably high reliability using the software [12].

**Figure 1:**
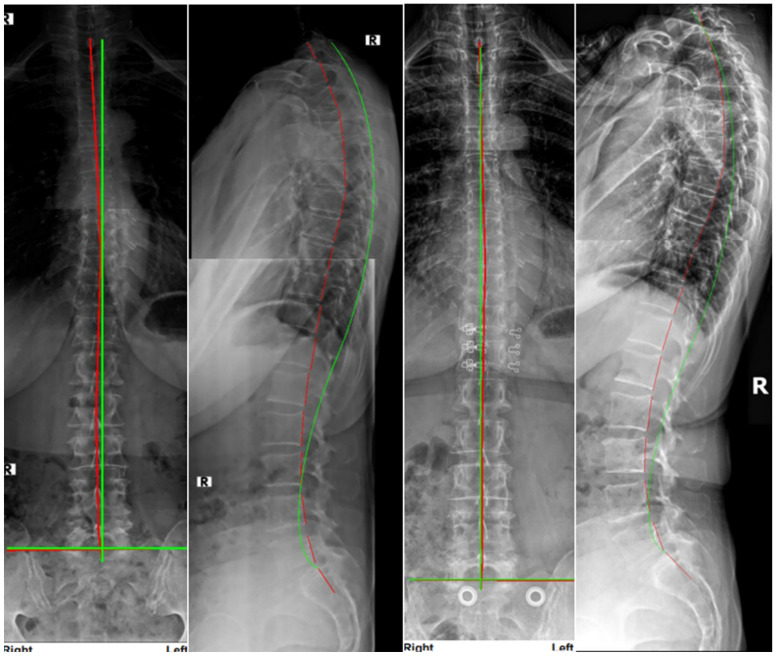
Patient A—Initial anterior–posterior thoraco-lumbar radiograph (**far-left**) and initial lateral thoraco-lumbar radiograph (**center-left**). Long-term follow-up radiographs (**center-right** and **far-right**). The red lines represent the patient’s biomechanical spine position along the posterior tangent of the vertebral bodies. The green lines represent ideal spine alignment. Slight right thoracic translation and slight right upper-thorax translation off of vertical plumb is visualized on the A–P film. The lateral/sagittal radiograph measured anterior thoracic translation with substantial flattening of the lumbar lordosis. Minimal change is seen in the A–P view beyond improvement in the lumbo-dorsal angle as measured with the Risser–Fergson method to determine center of mass of the vertebrae. The hypolordosis continues into the lower and mid-thoracic spine in the lateral view.

The baseline sagittal spine posterior vertebral body tangent measures absolute rotation angle (ARA) from the 2nd posterior body tangent to the 7th (ARA^C2–C7^). The ARA measured −0.6° (median is −34°, ideal is −42°, pain threshold cut-point is −20° [16]) (Figure 1). The AP cervical–thoracic X-ray demonstrated right lateral translation distance (T_X_^C2–C7^) relative to the true vertical of the lower cervical and upper thoracic spine measuring 15.9 mm (ideal is 0 mm [16]). AP thoracic and lumbar radiographs were unremarkable. There was a pointedly decreased sagittal curvature of the lumbar spine posterior body tangent measured from L1 to L5 (ARA^L1–L5^) at −21.8° (ideal is −40°, average range is from 35° to 45° [13]). This loss of lumbar lordosis was a further concern in the mismatch of her anatomically driven pelvic incidence angle of 83.5°, which indicated that the patient had a much deeper lordosis [17].

### 2.4. Patient B—History, Examination Results, Subjective and Objective Findings

A 43-year-old male (height 187.96 cm, weight 104.33 kg) reported a 7-year history of frequent and often severe LBP with a very recent episode of severe debilitating pain of insidious onset. He reported no known cause of his CLBP but reported it was made worse by activities of daily living (ADLs) such as sitting, standing, and walking. His reported pain and dysfunction were causing severe difficulty sleeping and he had tried chiropractic manipulation periodically before but there were no diagnostic tests performed, and the result of the manipulation was temporary pain relief with no improvement in function. NSAIDs were used and did not provide any long-term benefit. He reported a recent diagnosis of type 2 diabetes. The patient reported an active rural lifestyle where reduction in ADLs was having an impact on his quality of life and recreation including hunting, fishing, and hiking.

Detailed history, physical examination, orthopedic and neurological tests, and subjective and objective measures were acquired. Sensory dermatomal testing caused hypoesthesia at the S1 dermatome on the right. Standard lumbar orthopedic tests and range-of-motion pain stress testing were positive, and the patient was unable to perform any of the tests without an increase in low-back and buttock pain on the right. Visually assessed pain provocation range-of-motion (ROM) testing of the lumbar spine found very restricted lumbar flexion and extension with severe lumbar paraspinal musculature pain and radiating right buttock pain. Seated tests caused an increase in low back pain with radiation to the right lumbo-pelvic spine. The straight leg raiser (SLR) test was positive for low back and leg pain on the left. Cervical pain provocation ROM testing increased mild cervical pain with lateral cervical bending to the right side. Visual postural assessment found positive sagittal balance.

PROs were completed by the patient for multiple regions for spine pain and general health, dysfunction, and disability. The RODI score was 44%, indicating severe disability due to CLBP (Table 1). The SF-36 HRQoL short-form questionnaire to assess self-reported overall health found multiple deficiencies compared to norms and indicated that his pain and suffering were greatly reducing his QOL (Table 2). Worst measured deficiencies compared to normal on the SF36 were found in the physical (0/100), emotional (0/100), and energy/fatigue (30/100) categories, indicating an impact on QOL due to pain. Also significant was bodily pain (45/100), overall health perception (47/100), and social function (75/100) (Table 2).

Neck disability index measured 3/10 at baseline indicating mild neck pain, QVAS was assessed at baseline for the cervical, thoracic, and lumbar spine contribution to disability due to pain. The lumbar spine pain averaged 2/10 and the total pain composite score was reported as 30/100, indicating moderate impact of CLBP on patient QOL. The patient reported that this pain interfered with multiple normal ADLs such as sitting, walking, and bending.

### 2.5. Patient B Radiographic Findings

**Figure 2:**
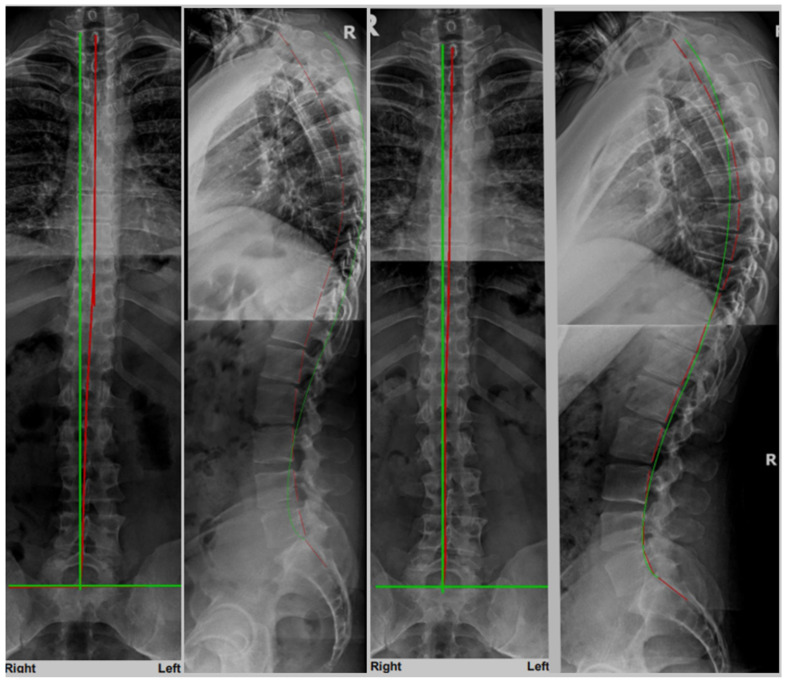
Patient B—Initial A–P and Lateral (**far-left** and **center-left**) and 26-month follow-up radiographic (**center-right** and **far-right**) examination revealed maintained sagittal balance and cervical ARA^T1–T12^ measuring 22.7°, or 3° compared to baseline and above neck pain cut-off of 20°. Coronal spinal alignment correction improvements toward normal continued with Tx^T1–T12^ measured only 2.2 mm from ideal (0.00 mm). Long-term follow-up found minimal forward head posture on the lateral cervical radiograph with C2–C7 translation measuring −22.7 mm, improved from 31.8 mm initially. Lateral lumbar biomechanical parameters measured via radiographs were well maintained at long-term follow-up with less than 2° overall difference from initial ARA^L1–L5^ and SBA (34.6° vs. initial 21.8°). Coronal and sagittal balance were well -preserved at 2-year follow-up on radiographs. The dotted red line represents the patient center of mass of the spine in the coronal plane, and the solid red line represents the patient’s sagittal balance. The green line represents a vertical plumb line in the A-P and the Harrison normal spine model in the sagittal plane.

The initial lateral cervical spine posterior body tangent ARA^C2–C7^ measured −17.5° (average is −34°, ideal is −42°, pain threshold is −20° [16]). AP cervical and thoracic radiographs were unremarkable. The most significant biomechanical abnormality was visualized on the lateral lumbar radiograph. There was a significantly decreased sagittal curvature using HPTM ARA^L1–L5^ measuring −22.6° (ideal is −40°, average range is from 35° to 45° [13,15]). Like patient A, the radiograph showed a mismatch of an anatomically driven pelvic incidence angle of 77.1°, which indicated that the patient had a much deeper lordosis in the range of L1–L5 [17] (Figure 2).

### 2.6. Treatment Protocols

Both patients agreed to participate in the multi-modal rehabilitation program intended to improve the measured abnormal spine and posture parameters and reduce pain and disability. This protocol has been previously described as necessary to include the three-pronged approach of postural exercises, traction, and postural manipulative therapeutics to achieve results similar to the prior RCTs and SROL studies. The patients received in-office treatment 36 times over 3 months (12 weeks) [18]. The patients received CBP^®^ Mirror Image^®^ (MI^®^) spinal/postural manipulations/adjustments using a chiropractic drop table mechanism to stimulate mechanoreceptors in the MI^®^ position. These MI^®^ adjustments position the patients in the inverse of the measured postural and spine parameter abnormalities. After the patient is properly positioned, the posture is then manipulated in the over-corrected posture.

**Figure 3:**
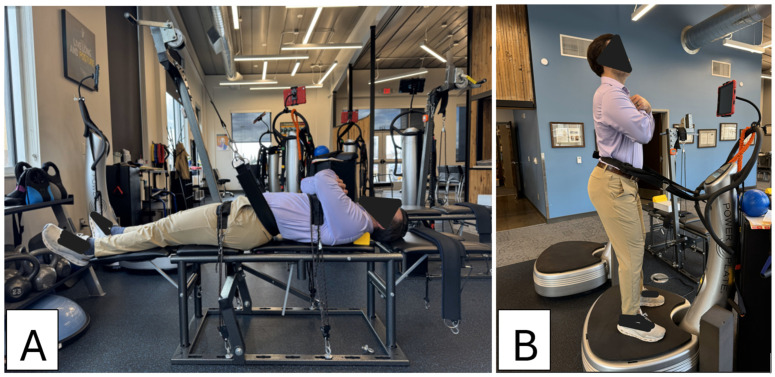
(**A**) Supine lumbar traction using the Universal Traction System^®^. The patient is placed supine with a restraining strap placed below the femur heads to allow pelvic flexion while a force vector from a pulling strap placed at approximately the L3/L4 disk plane line pulls posterior–anterior with a progressive increase in tension. A second restraining strap is placed in the lower ribcage to prevent anterior translation of the thorax. A small Denneroll cervical traction orthotic is placed in the lower neck to improve sagittal balance. (**B**) The Pro-Lordotic^TM^ lumbar exercise device is used on a Power Plate^®^. An elastic strap through the mid-lumbar spine is lengthened against extension resistance of the lumbar paraspinal muscles. The contraction is held for 15 s and performed for no longer than 10 min.

Both patients underwent MI^®^ traction using the Universal Traction Systems^®^ (UTS^®^) (Universal Traction Systems, Inc., Las Vegas, NV, USA) with the patients in a supine position on a flat bench using an anterior-to-posterior restraining strap holding the upper ribcage to the table under the arms. Additionally, another restraining strap was positioned at mid-thigh level to allow for increased pelvic flexion. Furthermore, a posterior-to-anterior (PA) static pull using a sturdy soft four-inch strap connected to a pully was positioned near L3-L4 through the lateral lumbar visualized disk plane line. A Denneroll^TM^ cervical traction orthotic (DCTO) was placed under the upper thoracic spine to address sagittal imbalance. The traction was applied beginning with 5 min and working up to 12 min per treatment in-office according to prior CBP^®^ lumbar extension traction (LET) protocol publications [19] (Figure 3). The patients also used the Circular Traction^®^ (Circular Traction, LLC. Huntington Beach, CA, USA) ambulatory traction device which fits around the lower torso and applies a static force across the lower lumbar spine to increase lordosis while walking in place on a Power Plate^®^ (Northbrook, IL, USA).

**Figure 4:**
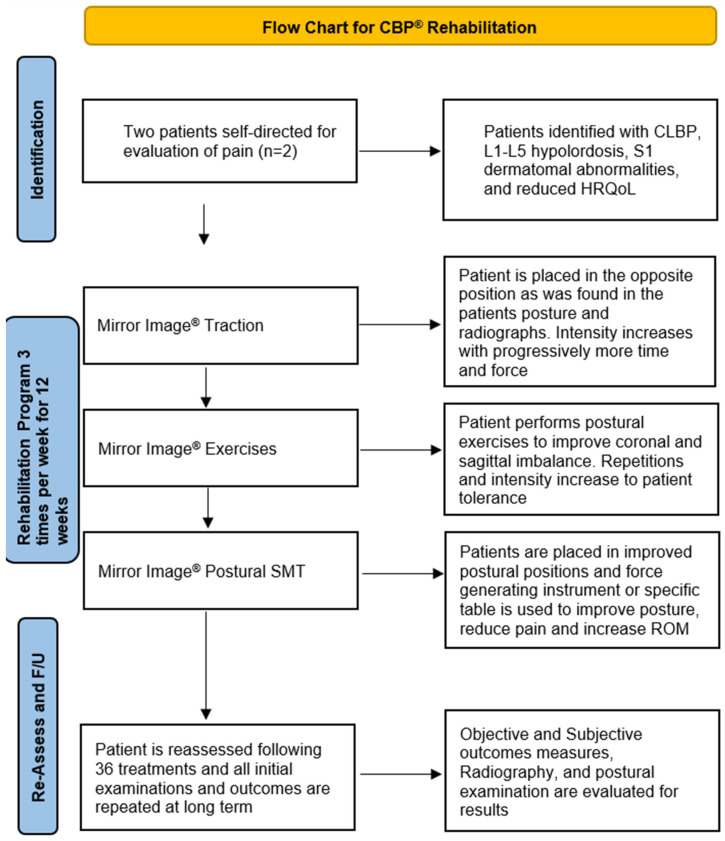
Flowchart of CBP^®^ rehabilitation protocol. The protocol involves traditional examination procedures to determine the candidacy of the patient for CBP^®^. The treatment program is individualized to match the abnormal postures and radiographic findings to a specific regiment of Mirror Image^®^ Postural adjustments, MI^®^ traction, and MI^®^ exercises.

MI^®^ exercises were performed on the Power Plate^®^ targeting lumbar extension positions for strengthening the lumbar paraspinal musculature using a slightly flexible strap for resistance located in the lumbar spine. The exercises were performed at ever-increasing tolerance to mild muscle soreness using the Pro-Lordotic Lumbar Exerciser^TM^ (Circular traction, LLC. Huntington Beach, CA, USA) (Figure 3B) on the Power Plate^®^ to address abnormal sagittal imbalance [20]. The patients were prescribed daily home care consisting of lumbar extension exercises held for no longer than 15 s and worked up to 50 repetitions per treatment. Similar traction protocols were followed, with the patient using the cervical and lumbar Dennerolls, beginning the traction for 2–5 min per session depending on tolerance and increasing to up to 15 min of traction per day. All three MI^®^ exercises, specific postural adjustments, and traction were completed on each visit by both patients. The rehabilitation protocols are presented in a flowchart (Figure 4).

## 3. Results

### 3.1. Post-Treatment Follow-Up Examination Findings

Following treatment, all baseline questions, tests, and objective questionnaires were repeated and compared to baseline findings. Exam results revealed that both patients reported significantly reduced CLBP, improved function, and reduced disability due to pain. Patient A self-reported a reduction in CLBP by 80%, IT band pain reduced 90%, cold hands and feet improved by 70%, constipation related to migraines reduced by 70%, neck flexibility improved by 70%, and migraines were significantly reduced in frequency and intensity, down to less than 1 per month. Orthopedic exams for patient A revealed very mild pain with hip joint standard ROM testing, all S1 hypoesthesia was resolved, maximal compression in the cervical spine was positive for very mild discomfort on the right, all ROM was within-normal-limits (WNL), and posture was improved visually. Patient B reported that all CLBP was no longer a problem and improved by 100%, neck stiffness was resolved by 100%, and all other initial symptoms were resolved. Patient B had no positive orthopedic tests, no hypoesthesia, all ROM was WNL, and posture was improved visually. Resolution of most baseline health conditions except the type 2 diabetes symptoms was reported.

### 3.2. Patient A Radiographic Results

Post-treatment radiographic examination revealed the following: minimally improved ARA^C2–C7^ measuring −5.8° (vs. −0.6° initially); Translation of C2 over C7 on the *z*-axis of the thorax (+T_Z_^H C2–C7^) measured 13.9 mm compared to initial 19.5 mm (vs. normal range of 0–15 mm); improved coronal thoracic translation −T_x_^T1–T12^ measuring −5.7 mm vs. initial 10.1 mm (vs. 0 mm normal); improved lumbar lordosis (ARA^L1–L5^) measuring −33.6° vs. initial −21.8 (vs. −40° ideal and pelvic incidence predicted −53°); improved +T_x_^T8–T12^ measuring 3.5 mm (vs. 15.2 mm); [21] (Figure 1).

A two-year long-term follow-up radiographic evaluation revealed improved thoracic kyphosis by 20% from baseline toward ideal overall ARA^T1–T12^ measuring 42.4°, or 0.5% from ideal angulation (40.0°). Long-term follow-up found near-normal forward head posture in the lateral and coronal spine and maintained sagittal balance. Lateral thoracic and lumbar biomechanical parameters measured via radiographs were well-maintained at long-term follow-up with less than 1° overall difference from initial ARA^L1–L5^ and SBA (45° vs. 44°). Coronal and sagittal balance were maintained at 2-year follow-up; however, L1–L5 lordosis had regressed to 26.4° and was still improved from baseline (Figure 1). All chief complaints were reported to be nearly resolved at 2-year long-term follow-up evaluation. There were no positive orthopedic or neurological tests at follow-up examination. The patient continued to use the Pro Lordotic™ neck exerciser at home 1–3 times per week for up to 10 min (Figure 3B) and the DCTO approximately 2–3 times per month. She was very pleased with the results of the treatment and the maintenance and continued improvements at 2-year follow-up. Her overall satisfaction was reported as very high. The improvements are consistent with prior RCT studies of the CBP^®^ protocols.

### 3.3. Patient A Patient Reported Outcome Results

Post-treatment RODI score was 12% (vs. 34%) indicating minimal disability due to low back pain following treatment. However, composite QVAS for the thoracic (3/100) and lumbar (10/100) regions was unchanged compared to baseline. Improved HDI composite at post-treatment measured 36/100 vs. 56/100 initial assessment. All post-treatment SF-36 scores showed improvements (Table 1) with the largest improvements in the categories of health perception (15 points), physical function (25 pts), emotional function (100 pts), social function (37 pts), mental health (36 pts), bodily pain (23 pts), and energy/fatigue function (35 pts).

Two-year RODI score of 8/100 indicated minimal disability from baseline (34%). Long-term post-treatment SF-36 scores showed maintained and improved HRQoL. Overall health perception improved to 35/100 points, physical function improved 15 points, physical and emotional function improved to ideal 100/100. Social function improved 37 points from baseline and bodily pain, and energy fatigue improved 33 and 40 points, respectively (Table 1 and Table 2).

### 3.4. Patient B Radiographic Results, Objective Outcome Measures, and Long-Term Findings

Patient B’s post-treatment cervical spine parameters were improved with ARA^C2–C7^ measuring −25.7° vs. −17.5° initially and improved beyond the pain angulation cut point of −20°); improved coronal thoracic translation T_x_^T1–T12^ and T_x_^T1–S1^ was consistent throughout the spine with minimal 1–4 mm and 1–5 mm movement toward normal; improved lumbar lordosis (ARA^L1–L5^) measuring −42.4° vs. initial −22.6° and it did regress in angulation at 2-year follow-up to 23.2° (vs. 40° ideal and pelvic incidence predicted at −51° from L1–L5); improved +T_x_^T12-S1^ measuring 8.7 mm (vs. 25.2 mm initially); improved SBA from 33.5° initially to an angulation of 48.9 (40° ideal and predicted 53° from pelvic incidence angle of 77.1° [21]).

Post-treatment SF-36 scores showed improvements in the categories of health perception (20 pts); physical ability which changed to 100/100 compared to 0/100; physical function which improved 10 pts; social function (37 pts); bodily pain (67 pts); and energy/fatigue function (25 pts) (Table 2). Post-treatment RODI score was improved to 12% (vs. 44% initially) indicating only mild disability due to CLBP following treatment. NDI normalized to 0/100. Composite QVAS for the lumbar region was improved to 10/100 compared to baseline 40/100. The two-year follow-up RODI score was 0/100, indicating entirely resolved CLBP disability from baseline (44%).

All subjective baseline patient symptoms that initially brought the patients seeking care were reported to be nearly resolved at 2-year follow-up examination. All initial physical and orthopedic tests from baseline were duplicated. There were no positive orthopedic or neurological tests found. The patients both continued to use the ProLordotic™ neck exerciser to tolerance at home 3–5 times per week throughout the post-treatment time period. They both reported a high level of satisfaction and were very satisfied with the short and long-term results of the treatment regimen.

## 4. Discussion

This case series describes observed changes in pain, disability indices, and select radiographic parameters in two patients with non-specific CLBP following a multimodal CBP^®^-based rehabilitation program. Improvements were maintained at approximately two-year follow-up despite minimal ongoing in-office care. Both patients were comparable in pathology: both presented with non-specific CLBP, similar radiographic hypolordosis (L1–L5 ≈ –22°), pelvic-incidence mismatch, right S1 dermatomal hypoesthesia, positive sagittal balance, and rural lifestyle barriers to frequent care. These observations are consistent with findings reported in prior studies of similar CBP^®^ multimodal approaches (additional references available upon request and the complete bibliography of the protocol is available at cbpnonprofit.com). Baseline measurements of the spine determine the specific patient position for MI^®^ exercises and uses increasing repetitions and sustained contractions to improve strength and postural balance of the musculature.

The results of these two patients are consistent with prior CBP^®^ studies of traction forces applied according to measured abnormal spine angulation [4,6,8,18,19]. The application of forces using radiographic parameters provided patient-specific guidance for the intervention. Although this case presents only two patients using the specific treatment regimen, the methods have been previously extensively studied, and the observations are consistent with those prior studies. This rehabilitation regimen targets abnormal spine and postural alignment by addressing musculature and ligamentous asymmetries. Numerous prior studies including 10 systematic reviews, 21 randomized controlled trials, seven non-randomized controlled trials, seven prospective cohort studies, and 178 prior case reports have been described in the biomedical literature. For the sake of brevity, and to avoid significant self-citation, additional references will be made available upon request. Baseline measurements of the spine determine the specific patient position for MI^®^ exercises and uses increasing repetitions and sustained contractions to improve strength and postural balance of the musculature. The exercises begin with minimal intensity and progress to positions being held for longer duration and increased repetitions. Abnormal posture causes musculature asymmetries and loads the frame abnormally and can lead to dysfunction and pain [22,23,24,25].

General spinal manipulative therapy (SMT) has been used to treat spine pain with variable results concerning disability and long-term benefits. This treatment involved moving the patient through cervical thoracic and lumbar ranges of motion with the intent to move through the physiological space and frequently into the para-physiological space causing joint cavitation. Non-specific chiropractic SMT and general SMT increases ROM and improves pain briefly; however, no known studies have repeatable long-term improvement in subjective/objective outcome measures [26]. This result is consistent with our two patients who received general SMT from chiropractors prior to this intervention without long-term reduction in symptoms or disability [27,28].

The MI^®^ manipulation used herein is different and unique from general SMT as each manipulation brings the patient toward improved sagittal/coronal balance. Reducing postural abnormalities has been shown to reduce abnormal biomechanical loading on soft tissues such as intervertebral disk and paraspinal ligaments. Improving posture function has been shown to improve latency and amplitude of conduction time in the central nervous system [29,30,31]. Abnormal postures reduce function in spinal musculature and impact efficiency of the brain regions involved with postural and balance control [32,33]. MI^®^ SMT involves setting the patient in the mathematical inverse of the spinal abnormalities and introducing a force via a drop-table mechanism aimed at creating neuroplastic changes in postural control areas in the central nervous system [34,35].

CBP^®^ MI^®^ traction aims to deform viscoelastic spinal ligaments and intervertebral disks and other non-contractile tissue, that due to poor elasticity, do not respond to muscular exercises or manipulative thrusts. These ligaments require longer time-dependent loads to cause biomechanical creep to impart normal spine curvatures in the sagittal plane and forces to straighten the spine in the coronal plane and the musculoskeletal ligamentous structures render them susceptible to injury under abnormal postural loads [36]. The traction force vector straps are employed according to the measured abnormal spine parameters and uses as-comfortable-as-possible positioning of the patient in standing, seated, supine, and prone positions.

The application causes ligaments to slowly creep the viscoelastic structures toward a more neutral and balanced biomechanical position. Prior studies have shown long-term improvement when the loads on the spine and postural muscles are improved or normalized toward models of coronal and sagittal balance from both conservative or surgical approach investigations to lessen the risk and consequences of ASD [37,38,39]. The results of these two patients are consistent with prior studies of traction forces applied according to measured abnormal spine angulation. The patient’s reported changes in QOL were found in conjunction with the positive subjective outcome measures and PRO’s post-treatment, and this spine alignment change was maintained at long-term 2-year follow-up. These methods of improving sagittal balance and cervical and lumbar lordosis have been shown to be repeatable and reliable to induce increases in lumbar and cervical lordosis toward a more ideal angulation in both conservative and surgical studies.

The application of forces using radiographic parameters is not unique to CBP^®^ rehabilitation; however, the body of literature supporting the use of simply acquired radiographs of the spine is growing. Using radiography for biomechanical assessment and treatment options further elucidates the “bio” portion of the currently reported studies of bio-psycho-social models of CLBP [40,41]. Improving the understanding of the individual patient biomechanics can aid treatment triage. The use of these radiographs and the measured spine parameters dictate the treatment protocol and this alteration in techniques could not take place if the biomechanical radiographs were absent such as when used in traditional SMT and physical therapy [41].

Although this case presents only two patients using the specific treatment regimen, the methods have been previously studied extensively, and the results of this case series are consistent with those prior studies. This reliable and repeatable spine mensuration software and machine learning application makes the application of the SMT vectors, the use of specific postural exercises, and the application of traction efficient and reliable with strong literature backing the use of AI mensuration [12,42]. Additionally, historical and recent studies show no negative effect of the use of diagnostic radiography and the outcomes of those with better diagnostic understanding are improved. Failure to adequately diagnose ASD can have significantly greater harm than the diagnostic radiation X-ray images needed for correct diagnosis and triage [43,44,45,46]. Best practice guidelines for the treatment of CLBP should incorporate as much evidence as possible for recommendations and case studies, and case series can offer unique alternative treatment protocols and novel, inexpensive regimes to broaden the treatment group and improve GBD by reducing the suffering and disability of CLBP patients. CBP^®^ protocols are intended to reduce pain, improve HRQoL, and the patients are continuously encouraged to increase homecare, health independence, and individual responsibility to improve posture with continued and increased home-based exercises and traction and not a reliance on the physician or therapist providing the care.

Future longer-term (5-year or 10-year) follow-up re-evaluations would validate long-term outcome results. Physicians must always take into consideration potential limitations of patients that are present outside the scope of the practitioner. The astute clinician will co-manage with other providers, including specialists in mental health disorders, addiction specialists and, in cases of severe degeneration, surgical co-management can benefit the patient [47,48,49]. The co-management strategy is especially important for psychological and mental health complications with CLBP [50]. These severe conditions may limit the benefit of these interventions and future studies should reflect these restrictions if they arise. The authors understand that this approach may not be appropriate for all spine condition patients and wish only to provide an interesting case series of two patients with similar conditions and results.

This report has important limitations inherent to a two-patient case series. There was no control group or comparator intervention, as is normal in case reporting. However, the protocol is based on prior RCTs with proper sample sizes and control groups. The multimodal nature of the program precludes determination of which elements contributed most to the observed changes. Other factors, including natural variation, adherence to home exercises, nonspecific effects, or regression to the mean, cannot be excluded. Generalizability is limited, and causality cannot be inferred. Larger prospective studies, randomized and controlled, are necessary before broader conclusions can be drawn. Notably, both patients resided in a rural area. The protocol’s transition to primarily home-based care after the initial in-office phase may have practical relevance for similar populations, though this requires confirmation in larger studies.

## 5. Conclusions

The results of the presented case series utilized structural, functional, and postural orthopedic and MI^®^ care to treat non-specific CLBP and disabling dysfunction. This CLBP caused decreased HRQoL and abnormal PROs and was unresponsive to prior chiropractic manipulation. Economical multi-modal treatments could provide options for treating CLBP and lessen side-effects and complications associated with pharmacologic and more invasive interventions such as surgery, and larger case series and controlled trials are necessary to draw definite conclusions. Directed and specific exercises, MI^®^ Postural SMT, and traction improved spinal alignment and health outcomes in two patients. This case series shows the need for additional research regarding treating spinal biomechanical disorders and tailoring rehabilitation protocols in patients with chronic non-specific spine pain.

## Figures and Tables

**Table 1:** Revised Oswestry Disability Index and Headache Disability Index results.

Patient A	RODI	HDI
23 November 2020	34/100	56/100
12 May 2021	12/100	36/100
7 July 2023	8/100	26/100
**Overall Change**	26/100	30/100
**Patient B**	**RODI**	**HDI**
5 August 2021	44/100	n/a
19 January 2022	12/100	n/a
17 August 2023	0/100	n/a
**Overall Change**	44/100	n/a

**Note**: **Left**: Revised Oswestry disability index (RODI). The RODI calculates the total percentage of disability of patients suffering with chronic low back pain: 0–20% represents minimal disability; 21–40% represents moderate disability; 41–60% represents severe disability; 61–100% represents total disability. **Right**: The headache disability index (HDI) measures the level of disability associated with headache: 0–20 represents minimal impact; 21–40 represents moderate impact; 41–60 represents significant impact; 61–100 represents severe, debilitating impact.

**Table 2:** Short-Form 36 questionnaire results.

Patient A	Health Perception	Physical Function	Physical Ability	Emotional Health	Social Function	Mental Health	Bodily Pain	Energy/Fatigue
23 November 2020	47	80	0	0	38	48	45	30
12 May 2021	67	95	100	100	88	84	68	65
7 July 2023	77	100	100	67	88	80	78	65
**Overall Change**	**30**	**20**	**100**	**67**	**50**	**32**	**33**	**35**
**Patient B**	**Health Perception**	**Physical Function**	**Physical Ability**	**Emotional Health**	**Social Function**	**Mental Health**	**Bodily Pain**	**Energy/Fatigue**
5 August 2021	62	85	0	100	63	92	23	50
29 January 2022	72	100	100	100	100	92	100	90
17 August 2023	97	100	100	100	100	92	100	90
**Overall Change**	**3** **5**	**15**	**100**	**0**	**37**	**0**	**77**	**30**

**Note**: The Short-Form 36 (SF36) health status questionnaire calculates the overall function of multiple health parameters. Patient A is reported in the upper three rows, and Patient B is reported in the lower three rows with overall change reported below each category. The form calculates the functional levels for the patient where 0 is no function and 100 is perfect function. The normative expected averages for the patients based on age are Health Perception (72), Physical Functioning (84), Role-Physical (81), Role-Emotional (81), Social Functioning (83), Bodily Pain (75), Mental Health (75), Energy/Fatigue (61).

## Data Availability

The data presented in this study are available on request from the corresponding author due to privacy and ethical restrictions.
